# Over 3-Years Follow-Up of a Case of Primary Biliary Cholangitis With Autoimmune Hyperthyroidism: Factors Associated With Liver Dysfunction

**DOI:** 10.7759/cureus.32941

**Published:** 2022-12-25

**Authors:** Takuro Sato, Ichiro Kumagai, Kazuyuki Suzuki, Keisuke Kakisaka, Koichi Abe

**Affiliations:** 1 Gastroenterology, Morioka Municipal Hospital, Morioka, JPN; 2 Gastroenterology and Hepatology, Iwate Medical University, Yahaba-cho, JPN; 3 Gastroenterology and Hepatology, Iwate Medical University, Yahaba-cho, JPN; 4 Gastroenterology, Abe Saien Clinic, Morioka, JPN

**Keywords:** drug-induced liver injury (dili), graves´disease, autoimmune hyperthyroidism, extrahepatic manifestation, primary biliary cholangitis

## Abstract

We present a rare case of a 50-year-old woman with simultaneous primary biliary cholangitis (PBC) and autoimmune hyperthyroidism in which the factors associated with liver dysfunction were evaluated over a 3-year follow-up period. Although serum thyroid hormone levels improved after the administration of thiamazole, thyroid dysfunction was not directly associated with liver dysfunction. During the follow-up period, anti-hyperlipidemic drug therapy and eradication therapy for *Helicobacter pylori* (*HP*) infection caused transient elevation of serum transaminase levels. It should be recognized that serum liver enzyme levels might be affected by various factors, including the therapies for the many complications of PBC.

## Introduction

Primary biliary cholangitis (PBC), formerly known as primary biliary cirrhosis, is a chronic cholestatic and progressive liver disease caused by an autoimmune mechanism [1-3]. The histological features of PBC are characterized by the selective and progressive destruction of intrahepatic small to medium-sized bile ducts by inflammatory cells (mainly lymphocytes and plasma cells), leading to liver fibrosis and finally liver cirrhosis [4]. PBC has often been associated with many extrahepatic autoimmune diseases such as rheumatological disorders including Sjögren’s syndrome and rheumatoid arthritis and endocrine diseases, either simultaneously or sequentially [5-9]. Among endocrine disorders, Hashimoto’s disease with hypothyroidism has been commonly observed, but hyperthyroidism related to autoimmune thyroiditis (Graves’ disease) has been very rare in cases with PBC [10-16].

In PBC, levels of serum liver enzymes such as alkaline phosphatase (ALP) and γ-glutamyltranspeptidase (γGTP) are characteristically elevated, whereas the levels of serum transaminases, aspartate aminotransferase (AST) and alanine aminotransferase (ALT), are slightly or mildly elevated. Furthermore, PBC patients with autoimmune hepatitis (AIH) tend to have high serum AST and ALT levels [17,18]. On the other hand, thyroid dysfunction (hypothyroidism or hyperthyroidism) due to thyroid diseases has also been closely associated with the elevation of serum liver enzymes (liver injury) [19-21]. In addition, anti-thyroid drugs and/or some medications for other complications often induce liver injury because of their hepatotoxicity [22,23]. Therefore, it is very important to evaluate whether the abnormalities of serum liver enzymes are caused by PBC itself or secondary factors, including therapies for the many complications of PBC.

A case of a female PBC patient with simultaneous autoimmune hyperthyroidism whose liver and thyroid functions were evaluated for a period of over 3 years is presented. In this case, although thyroid function finally normalized, and both serum anti-thyroglobulin antibody (TgAb) and anti-thyroid peroxidase antibody (anti-TPOAb) both became negative due to thiamazole administration, the levels of serum liver enzymes, in particular serum AST and ALT levels, fluctuated despite treatment with ursodeoxycholic acid (UDCA). Serum AST and ALT levels were also transiently elevated after treatment for hyperlipidemia and eradication therapy for *Helicobacter pylori (HP)* infection. This case may provide useful information regarding the relationship between liver and thyroid dysfunctions during long-term follow-up.

## Case presentation

A 50-year-old woman with systemic pruritus visited a dermatology outpatient clinic in late April 2018 and received drug treatment. As she had symptoms of a common cold (rhinorrhea, cough, and sputum without fever) in the middle of May 2018, she visited the same clinic again and was found to have liver dysfunction in late May 2018. Thus, she visited another outpatient clinic and received injection therapy with a glycyrrhizin-containing product (Stronger Neo-Minophagen C; SNMC, Minophagen Pharmaceutical Co. Ltd., Tokyo, Japan). However, because no improvement in liver function was observed, she was referred to our hospital and admitted in early June 2018. Her medical history included a Caesarean section without blood transfusion at the age of 21 years. She also had been diagnosed with atopic dermatitis at the age of 20 years. Furthermore, she had been diagnosed with Epstein-Barr virus (EBV) infection four years earlier (details unknown). Alcohol intake was about 35 grams per day twice a week for about 30 years. On her admission physical examination, her height, weight, and body mass index were 160 cm, 47 kg, and 18.4 kg/m2, respectively. Her temperature was 35.6 °C. Her peripheral blood pressure was 122/79 mmHg, and her pulse rate was 93 beats/min. On chest examination, there were no crackles over the lung fields, and heart sounds were normal, with no murmurs. Although jaundice, skin eruption, systemic lymphadenopathy, ascites, and edema of the legs were absent, the soft liver was palpable about 3 cm below the costal margin in the epigastrium, but the spleen was not palpable.

**Figure 1:**
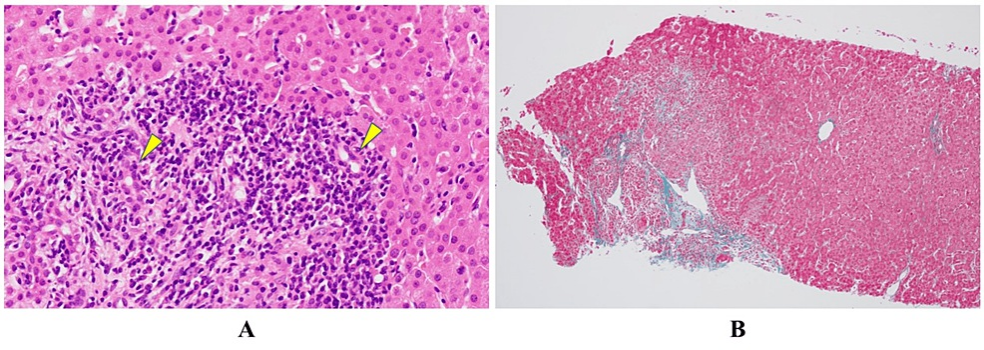
Histological findings (A) Hematoxylin and eosin staining (400×). (B) Elastica-Masson staining (100×). Pathological findings of a liver specimen obtained by needle biopsy. Interlobular bile duct destruction with infiltration of lymphocytes is seen in the enlarged portal area (arrow). Extended fibrosis is not seen.

Table 1 shows the laboratory data on admission. The red blood cell (RBC) count was within the normal range, but the hemoglobin (Hb) concentration and mean corpuscular hemoglobin (MCV) were decreased, whereas the platelet count was slightly increased. Serum liver enzyme (aspartate aminotransferase (AST), alanine aminotransferase (ALT), alkaline phosphatase (ALP), and γ-glutamyltranspeptidase (γGTP)) levels were significantly elevated. The serum total-bilirubin (T-Bil) level was slightly elevated, but prothrombin time activity was normal. The serum immunoglobulin (Ig) M concentration was significantly elevated, whereas IgA and IgG concentrations were normal. The serum antinuclear antibody (ANA) titer and anti-mitochondrial antibody M2 (AMA-M2) were both positive. Viral hepatitis markers (hepatitis B and C) were negative. Serum immunoglobulin M-class anti-hepatitis A antibody and immunoglobulin A-class anti-hepatitis E antibody were not examined as she had no history of drinking unsterilized water and no intake of raw or undercooked meat/offal from livestock or wild animals, and she also had no history of traveling abroad to hepatitis A virus and hepatitis E virus endemic areas, such as Southeast Asia. Of the serial markers of EBV and cytomegalovirus (CMV) infections, anti-EBV capsid antigen (EB-VCA) IgG, EB-VCA IgM, anti-CMV IgG, and anti-CMV IgM were positive, but anti-EB nuclear antigen (anti-EBNA) was not. However, the absence of typical signs, such as fever, pharyngitis, tonsillitis, and cervical axillary lymphadenopathy, indicated that infections with these viruses were not observed throughout the clinical course. Surprisingly, the thyroid-stimulating hormone (TSH) level was low, whereas free thyroid hormone 3 (FT3) and free thyroid hormone 4 (FT4) levels were considerably elevated. In addition, TgAb and anti-TPOAb titers were both clearly positive. Cervical sonography was not performed on admission, and she had no characteristic signs and/or symptoms of hyperthyroidism such as goiter, sweating, tachycardia, tremor, and weight loss. Abdominal sonography and computed tomography showed no findings indicating liver cirrhosis, no abnormal dilation of intrahepatic and extrahepatic bile ducts, and no gallbladder stones. Thus, a liver biopsy was first performed under abdominal sonography guidance to confirm the diagnosis of liver injury before treatment in the middle of May 2018. On histological examination of the biopsy specimen, there were dense inflammatory infiltrates (lymphocytes, plasma cells, and eosinophils), granulomas were seen in the enlarged portal area, and destructive interlobular bile ducts with infiltration of lymphocytes were seen, but there was no extended fibrosis (Figure 1). These histological features were compatible with primary biliary cholangitis (PBC) at Scheuer Stage II. Moreover, staining for CMV in liver tissue was negative. She was finally diagnosed with symptomatic PBC and asymptomatic hyperthyroidism due to autoimmune thyroiditis.

**Table 1:** Laboratory data on admission WBC: white blood cells, Neut: neutrophils, Lymp: lymphocytes, Mono: monocytes, Eosi: eosinophils, RBC: red blood cells, Hb: hemoglobin, Ht: hematocrit, Plt: platelets, PT: prothrombin time-international normalized ratio, TP: total protein, Alb: albumin, T-Bil: total bilirubin, D-Bil: direct bilirubin, AST: aspartate aminotransferase, ALT: alanine aminotransferase, LDH: lactic dehydrogenase, ALP: alkaline phosphatase, γ-GTP: gamma-glutamyl transpeptidase, BUN: blood urea nitrogen, Cre: creatinine, CRP: C-reactive protein, TSH: thyroid stimulating hormone, FT: free triiodothyronine, ANA: anti-nuclear antigen, AMA-M2: anti-mitochondrial M2 antibody, HBsAg: hepatitis B surface antigen, HCVAb: hepatitis C virus antibody, Ig: immunoglobulin, Tg: thyroglobulin, TPO: thyroid peroxidase

Complete blood count		
WBC	4500	µL
Neut	61.6	%
Lymp	10.8	%
Mono	3.4	%
Eosi	2.1	%
RBC	4.72×10^6^	µL
Hb	9.9	g/dL
Ht	31.7	%
Plt	462×10^3^	µL
Coagulation studies		
PT	>100	%
Blood chemistry		
TP	8.3	g/dL
Alb	3.3	g/dL
T-Bil	1.3	mg/dL
D-Bil	0.8	mg/dL
AST	183	U/L
ALT	195	U/L
LDH	300	U/L
ALP(IF)	2796	U/L
γ-GTP	713	U/L
BUN	13.2	mg/dL
Cre	0.40	mg/dL
CRP	0.18	mg/dL
TSH	<0.005	µIU/mL
FT3	7.72	pg/mL
FT4	2.20	ng/dL
Serological tests		
ANA	×640	
AMA-M2	>=400	
HBsAg	(-)	
HCVAb	(-)	
IgG-EBV VCA Ab	(+)	
IgM-EBV VCA Ab	(+)	
EB-EBNA Ab	(-)	
IgG-CMV EIA Ab	(+)	
IgM-CMV EIA Ab	(+)	
IgG	1566	mg/dL
IgM	716	mg/dL
Anti-TSH-R Ab	18.5	%
Anti-Tg Ab	301	U/mL
Anti-TPO Ab	56.8	U/mL

**Figure 2:**
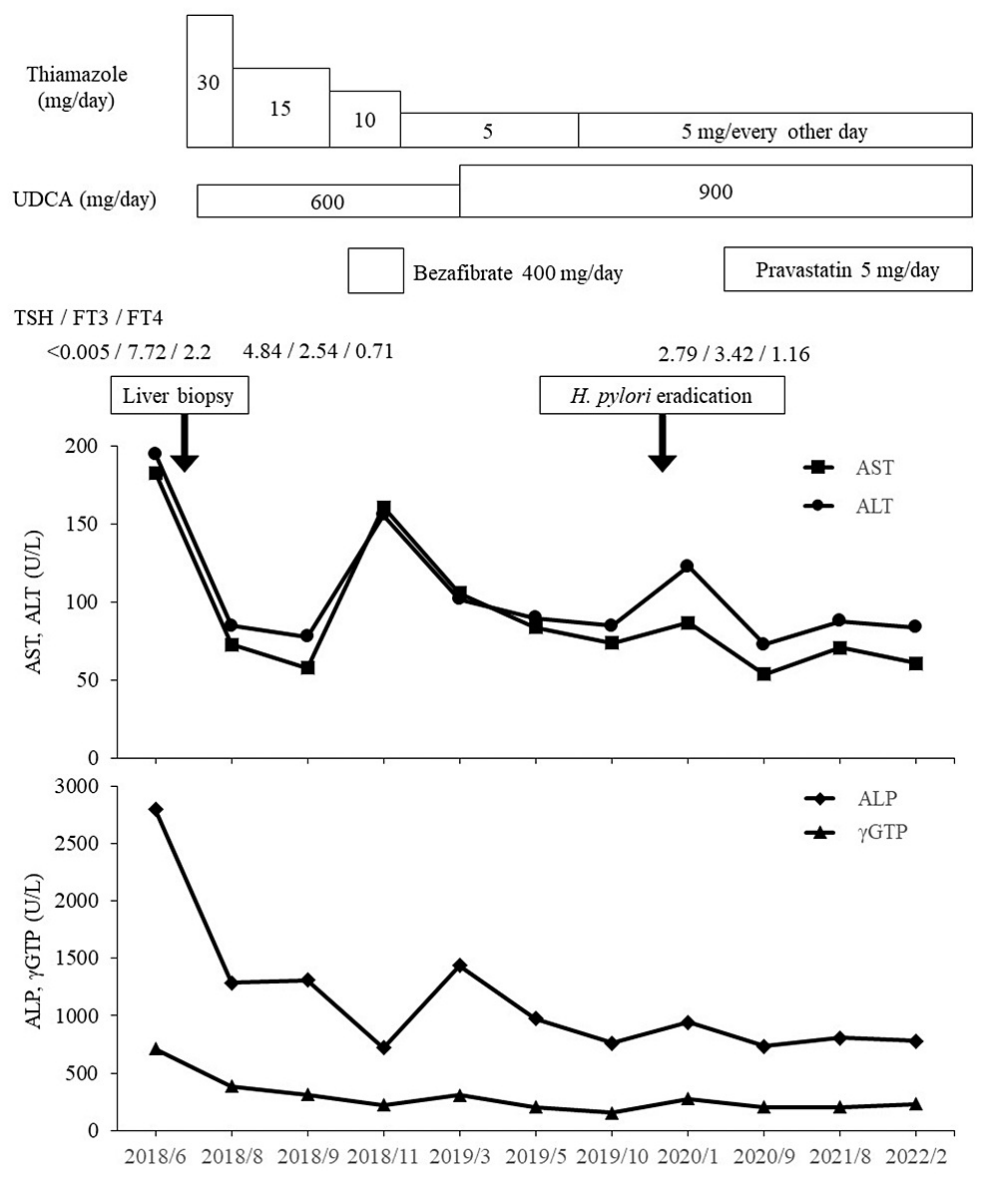
Changes in serum liver function tests during the course of the illness UDCA: ursodeoxycholic acid, TSH: thyroid stimulating hormone, FT: free triiodothyronine, AST: aspartate aminotransferase, ALT: alanine aminotransferase, ALP: alkaline phosphatase, γGTP: gamma-glutamyltranspeptidase

Figure 2 shows her clinical course, including medical treatments and serial changes in serum liver enzyme levels and thyroid hormone concentrations. Thiamazole (initial dosage 30 mg/day) had been administered from the day after the liver biopsy, and its dosage was gradually reduced based on the changes in serum thyroid hormone concentrations. The concentrations of TSH, FT3, and FT4 decreased gradually and finally normalized. Both serum TgAb and anti-TPOAb became undetectable 168 days after admission. Ursodeoxycholic acid (UDCA; initial dosage 600 mg/day) was administered since the middle of May 2018. As serum ALP and γGTP levels did not improve markedly after the administration of UDCA, the dosage of UDCA was increased to 900 mg/day from early March 2019. Bezafibrate (Bezatol SR; Kissei Pharmaceutical Co. Ltd., Nagano, Japan), an anti-hyperlipidemic drug, was added for hyperlipidemia. However, because her serum AST and ALT levels were elevated, this drug was stopped immediately. In addition, since she complained of epigastric pain in November 2019, endoscopy of the upper gastrointestinal tract was performed, confirming a gastric ulcer (active stage) in the midbody of the stomach with *Helicobacter pylori (HP)* infection. After *HP* eradication therapy, the serum AST level was transiently elevated. On the other hand, since the cause of her anemia was iron deficiency which was associated with a large myoma uteri, oral administration of sodium ferrous citrate (100 mg/day) was performed from late October 2019 to the middle of January 2020, but this drug did not affect the serum transaminase levels.

Serum EBV and CMV markers, except EBNA-antibody, were positive in this patient, but she had no characteristic signs and symptoms associated with these viral infections. Although the serum CMV-IgM antibody became negative, EB-VCA IgM antibody was unfortunately not measured during the course of her illness.

## Discussion

The case of a woman with primary biliary cholangitis (PBC) who had asymptomatic autoimmune hyperthyroidism was presented, and the factors associated with liver dysfunction (mainly abnormalities of liver enzymes) were evaluated based on long-term follow-up over 3 years. Although PBC is clinically classified into symptomatic and asymptomatic types, its diagnosis is relatively easy in typical cases showing high levels of serum alkaline phosphatase (ALP) and γ-glutamyltranspeptidase (γGTP) with positive serum anti-mitochondrial antibody (AMA) (and/or AMA-M2) [1-3]. Patients with symptomatic PBC usually have jaundice, systemic pruritus, and general fatigue. Since the present patient had a long-standing history of mild systemic pruritus, it was considered that she had both allergic dermatitis and PBC. Because PBC is a chronic and cholestatic liver disease caused by an autoimmune mechanism, PBC is often associated with many extrahepatic disorders, including thyroid diseases. According to a summary in a previous report, the prevalence of thyroid diseases with PBC is 9-23.6% [6]. Among the thyroid diseases, in general, Hashimoto’s disease showing hypothyroidism is common, whereas Graves’ disease showing hyperthyroidism is extremely rare. Several studies have evaluated the prevalence of thyroid diseases in PBC patients. An Italian group reported that 150 (16.3%) of a total of 921 PBC cases had thyroid diseases, of which 94 (10.2%) had Hashimoto’s disease, and 15 (1.6%) had hyperthyroidism (Graves’ disease) [5]. Interestingly, they found that the prevalence of Graves’ disease differed between regions (Padua and Barcelona). Furthermore, Efe et al. reported that, of 1,554 PBC cases with extrahepatic diseases in 20 international centers in Europe, the United State of America, and Canada, 166 (10.6%) had coexistent autoimmune thyroid disease: 141 (9.07%) had Hashimoto’s disease; 24 (1.54%) had Graves’ disease [7]. Furthermore, Liu et al. reported that the prevalence of Graves’ disease was 0.6% (3 cases), whereas that of Hashimoto’s disease was 0.2% (1 case) among 505 Chinese patients with PBC [8]. Recently, the prevalence of many extrahepatic autoimmune diseases associated with PBC has also been reported in Japanese patients [9]. In this report, although Hashimoto’s disease was seen in 39 (7%) of 524 cases, the prevalence of hyperthyroidism was unfortunately not described. Thus, we investigated the prevalence of PBC with autoimmune thyroid diseases in our two institutes (Morioka Municipal Hospital and Iwate Medical University Hospital) since 2015. Hyperthyroidism (Graves’ disease) was seen in only 1 (the present case) of 87 cases (1.1%) and 1 of 77 cases (1.3%), respectively, whereas Hashimoto’s disease was seen in 1 of 87 cases (1.1%) and 5 of 77 cases (6.5%), respectively (unpublished data). The prevalence of Graves’ disease associated with PBC differed among regions [5]. Furthermore, as Liu et al. showed that the cumulative incidence rate of autoimmune diseases including thyroid diseases (the rate of Graves’ disease was unknown) during 30-year follow-up increased from 4.8% to 16% in 150 Taiwanese PBC patients treated with ursodeoxycholic acid (UDCA) from a 30-year cohort study [8], further study is needed to clarify the true prevalence of Graves’ disease in PBC patients in each country.

Next, PubMed was searched for previous PBC cases with hyperthyroidism in the English literature from 1982 to 2020 (although the term “Graves’ disease” and “hyperthyroidism” are not synonymous). As shown in Table 2, eight cases (7 female, 1 male) including the present case were identified. Although the times of onset of PBC and Graves’ disease differed among these cases, only two cases including the present case showed simultaneous onset. Severe jaundice was seen in one case. Furthermore, there were three PBC cases with Graves’ disease published in Japanese journals (data not shown; only the abstracts are in English).

**Table 2:** Clinical features of primary biliary cholangitis with autoimmune hyperthyroidism (Graves’ disease) PBC: primary biliary cholangitis, GD: Graves’ disease, f: female, m: male, CREST syndrome: Calcinosis-Raynaud phenomenon-esophageal involvement-sclerodactyly-telangiectasia syndrome, AIH: autoimmune hepatitis

Case	Age /sex	Initial disease and time for PBC and GD association (clinical characteristics)	Complications of autoimmune diseases	Reference. NO.
1	48/f	GD, 3 years	None	[10]
2	54/f	Simultaneously	Sjögren’s syndrome + Systemic sclerosis	[11]
3	61/f	PBC, 6 years	Sjögren’s syndrome ＋CREST syndrome	[12]
4	51/f	GD, 5 years	None	[13]
5	50/f	GD, 1 year (severe jaundice, but reversible)	None	[14]
6	54/f	PBC, 5 years	Autoimmune hepatitis + Immune thrombocytopenia	[15]
7	62/m	GD, 4 years	None	[16]
8	50/f	Simultaneously	s/o PBC-AIH overlap syndrome	Present case

The present case showed high serum antinuclear antibody (ANA) titers, but the findings on histological examination did not clearly support primary biliary cholangitis and autoimmune hepatitis (PBC-AIH) overlap syndrome [18,19]. It will be necessary to carefully observe the disease course in the future.

The elevation of serum biliary tract enzymes such as alkaline phosphatase (ALP) and γ-glutamyltranspeptidase (γGTP) shows the characteristic pattern of liver function tests whether the PBC is symptomatic or asymptomatic. On the other hand, serum transaminases (aspartate aminotransferase (AST) and alanine aminotransferase (ALT)) show slight elevation, but these levels are affected by the following factors: 1) with or without autoimmune hepatitis (AIH); 2) the existence of hormonal abnormalities related to steroid and thyroid hormones inducing liver injury; and 3) administration of additional hepatotoxic drugs. In the present case, it was very difficult to evaluate whether the initial elevations of AST and ALT were associated with the PBC itself or secondary to thyroid dysfunction. As shown in Figure 2, high levels of serum transaminases in the initial stage (before and after the early admission period) and the levels of serum transaminases during follow-up were not directly associated with hyperthyroidism and the administration of anti-thyroid drugs because the levels of serum transaminases fluctuated despite thyroid function normalization. Before admission to our hospital, she received intravenous administration of SNMC for 6 days, although the exacerbation of live injury caused by this drug cannot completely rule out. Furthermore, serum transaminase levels were transiently elevated after administration of an anti-hyperlipidemic drug and Helicobacter pylori (HP) eradication therapy including two antibiotics that can cause drug-induced liver injury. Therefore, although we finally confirmed the cause of secondary liver injury during the course of illness, the present case suggests that serum liver enzyme levels may be affected by various factors, including the therapies for the many complications of PBC.

Recently, it has been reported that the reactivation of Epstein-Barr virus (EBV) is closely associated with thyroid-stimulating hormone receptor antibody (TRAb) production, resulting in the development of Graves’ disease [24]. The present case had a history of EBV infection 4 years earlier (details unknown), and among the serial markers of EBV and cytomegalovirus (CMV) infection, anti-EBV immunoglobulin (Ig) G, anti-EBV IgM, anti-CMV IgG, and anti-CMV IgM were positive, but anti-EB nuclear antigen (anti-EBNA) was not. These data suggest that both EBV and CMV infections and/or reactivation of these viruses could have occurred in the early stage, although typical clinical manifestations associated with infections by these viruses were not recognized. Regarding the serial changes in these EBV and CMV markers during the course of illness, anti-CMV IgM became negative, but anti-EBV IgM was not examined. It is necessary to evaluate the relationship between the serum TRAb titer and the clinical status of PBC with Graves’ disease in the future.

Finally, multiple genetic factors including human leukocyte antigen (HLA) region polymorphism have been associated with susceptibility to PBC and Graves’ disease [25]. It has been reported that the DRB1*08:03 allele and the DRB*08:03-DQB1:06:01 haplotype are associated with PBC in Japanese patients [26]. In addition, Suzuki et al. reported a male case with DRB1*08:02 [16]. Although HLA typing could not be performed in the present case, a further study of genetic factors is needed in Japanese PBC patients with thyroid disease.

## Conclusions

We presented a rare case of concurrent primary biliary cholangitis (PBC) and autoimmune hyperthyroidism and evaluated factors associated between liver and thyroid dysfunction over a 3-year period. In this case, thyroid dysfunction was not associated with direct liver dysfunction. During the follow-up period, transient elevation of serum transaminase (aspartate aminotransferase (AST) and alanine aminotransferase (ALT)) levels has been observed after treatments for hyperlipidemia and Helicobacter pylori (HP) infection, respectively, and the real cause was not clear (administration of anti-hyperlipidemic drug and/or antibiotics-induced liver injury). In conclusion, the relationship between liver and thyroid function in PBC patients with hyperthyroidism should be carefully evaluated during long-term follow-up because serum liver enzyme levels can be influenced by a variety of factors including treatments for many complications of PBC.
